# Supplementary material for: The enpp4 ectonucleotidase regulates kidney patterning signalling networks in Xenopus embryos
Source: Commun Biol. 2021 Oct 7;4:1158. doi: 10.1038/s42003-021-02688-9 (PMC8497618; doi:10.1038/s42003-021-02688-9)
Supplement: Supplementary file 3 — Reporting Summary [file 42003_2021_2688_MOESM3_ESM.pdf]

nature research

Corresponding author(s): Masse Karine - COMMSBIO-21-0713-A

Last updated by author(s): Aug 30, 2021

Reporting Summary

Nature Research wishes to improve the reproducibility of the work that we publish. This form provides structure for consistency and transparency in reporting. For further information on Nature Research policies, see our Editorial Policies and the Editorial Policy Checklist.

Statistics

For all statistical analyses, confirm that the following items are present in the figure legend, table legend, main text, or Methods section.

n/a	Confirmed	
☐	☒	The exact sample size (n) for each experimental group/condition, given as a discrete number and unit of measurement
☐	☒	A statement on whether measurements were taken from distinct samples or whether the same sample was measured repeatedly
☐	☒	The statistical test(s) used AND whether they are one- or two-sided<br>*Only common tests should be described solely by name; describe more complex techniques in the Methods section.*
☒	☐	A description of all covariates tested
☐	☒	A description of any assumptions or corrections, such as tests of normality and adjustment for multiple comparisons
☒	☐	A full description of the statistical parameters including central tendency (e.g. means) or other basic estimates (e.g. regression coefficient) AND variation (e.g. standard deviation) or associated estimates of uncertainty (e.g. confidence intervals)
☐	☒	For null hypothesis testing, the test statistic (e.g. F, t, r) with confidence intervals, effect sizes, degrees of freedom and P value noted<br>*Give P values as exact values whenever suitable.*
☒	☐	For Bayesian analysis, information on the choice of priors and Markov chain Monte Carlo settings
☒	☐	For hierarchical and complex designs, identification of the appropriate level for tests and full reporting of outcomes
☒	☐	Estimates of effect sizes (e.g. Cohen's d, Pearson's r), indicating how they were calculated

Our web collection on statistics for biologists contains articles on many of the points above.

Software and code

Policy information about availability of computer code

Data collection	No software was used.
Data analysis	No software was used

For manuscripts utilizing custom algorithms or software that are central to the research but not yet described in published literature, software must be made available to editors and reviewers. We strongly encourage code deposition in a community repository (e.g. GitHub). See the Nature Research guidelines for submitting code & software for further information.

Data

Policy information about availability of data

All manuscripts must include a data availability statement. This statement should provide the following information, where applicable:

- Accession codes, unique identifiers, or web links for publicly available datasets
- A list of figures that have associated raw data
- A description of any restrictions on data availability

The raw data are given in the Supplementary Information section, in the Supplementary Table 1A, 2A, 3A, 4 and 5A.

Field-specific reporting

Life sciences study design

All studies must disclose on these points even when the disclosure is negative.

Sample size	No sample-size calculation was carried out. Numbers of scored embryos for each experiment (indicated in the supplementary tables) are similar with those analysed in similar published studies and allowed to carry out statiscal analyses (Chi2 or Fisher)
Data exclusions	The criteria for excluding embryos (wrong targeting or abnormal kidney on the uninjected side) are also indicated in the Method Sections
Replication	The number of experiments (embryo batch=biological replication) carried out is indicated in the Method Sections.
Randomization	embryos were randomly includeedd in experimental groups
Blinding	The scoring of the embryos was performed blind-coded. Embryos were fixed in tubes identified by code (colours identification, no mention of dates or conditions) and experiments and analysis were carried out using this code.

Reporting for specific materials, systems and methods

We require information from authors about some types of materials, experimental systems and methods used in many studies. Here, indicate whether each material, system or method listed is relevant to your study. If you are not sure if a list item applies to your research, read the appropriate section before selecting a response.

Materials & experimental systems

n/a	Involved in the study	
☐	☒	Antibodies
☒	☐	Eukaryotic cell lines
☒	☐	Palaeontology and archaeology
☐	☒	Animals and other organisms
☒	☐	Human research participants
☒	☐	Clinical data
☒	☐	Dual use research of concern

Methods

n/a	Involved in the study	
☒	☐	ChIP-seq
☒	☐	Flow cytometry
☒	☐	MRI-based neuroimaging

Antibodies

Antibodies used	3G8 and 4A6 antibodies (hybridomas) were made in our laboratory and were published in P. D. Vize, E. A. Jones, R. Pfister, Development of the Xenopus pronephric system. Dev Biol 171, 531-540 (1995).<br>Secondary antibodies were purchased from Sigma; Anti-Digoxigenin-AP antibody from Roche.
Validation	3G8 and 4A6 antibodies were validated and published in P. D. Vize, E. A. Jones, R. Pfister, Development of the Xenopus pronephric system. Dev Biol 171, 531-540 (1995).

Animals and other organisms

Policy information about studies involving animals; ARRIVE guidelines recommended for reporting animal research

Laboratory animals	Xenopus laevis males and females.
Wild animals	the study did not involved wild animals
Field-collected samples	The sudy did not involve samples collected in the field
Ethics oversight	The work was carried out under a UK Home Office-approved animal procedures project licence and approved by the University of Warwick Biological Ethics Committee

Note that full information on the approval of the study protocol must also be provided in the manuscript.
